# The Genetic Diversity of the Nguni Breed of African Cattle (*Bos* spp.): Complete Mitochondrial Genomes of Haplogroup T1

**DOI:** 10.1371/journal.pone.0071956

**Published:** 2013-08-19

**Authors:** K. Ann Horsburgh, Stefan Prost, Anna Gosling, Jo-Ann Stanton, Christy Rand, Elizabeth A. Matisoo-Smith

**Affiliations:** 1 Department of Anatomy, University of Otago, Dunedin, New Zealand; 2 School of Geography, Archaeology and Environmental Studies, University of the Witwatersrand, Gauteng Province, South Africa; 3 Department of Integrative Biology, University of California, Berkeley, California, United States of America; 4 Allan Wilson Centre for Molecular Ecology and Evolution, University of Otago, Dunedin, New Zealand; Durham University, United Kingdom

## Abstract

Domesticated cattle were commonplace in northern Africa by about 7,000 years ago. Archaeological evidence, however, suggests they were not established in southern Africa until much later, no earlier than 2,000 years ago. Genetic reconstructions have started to shed light on the movement of African cattle, but efforts have been frustrated by a lack of data south of Ethiopia and the nature of the mitochondrial haplogroup T1 which is almost fixed across the continent. We sequenced 35 complete mitochondrial genomes from a South African herd of Nguni cattle, a breed historically associated with Bantu speaking farmers who were among the first to bring cattle to southern Africa. As expected, all individuals in the study were found to be members of haplogroup T1. Only half of the sub-haplogroups of T1 (T1a-T1f) are represented in our sample and the overwhelming majority (94%) in this study belong to subhaplogroup T1b. A previous study of African cattle found frequencies of T1b of 27% in Egypt and 69% in Ethiopia. These results are consistent with serial multiple founder effects significantly shaping the gene pool as cattle were moved from north to south across the continent. Interestingly, these mitochondrial data give no indication that the impacts of the founder effects were ameliorated by gene flow from recently introduced Indian cattle breeds.

## Introduction

### Cattle Domestication

Domestic cattle have their ancestry among wild auroch (*Bos primigenius*), a taxon naturally distributed across northern Africa, Eurasia and India which became extinct in the 17^th^ century [1]. Modern domesticated cattle comprise two distinct, though completely interfertile groups: taurine and indicine cattle. Taurine cattle (*Bos taurus*) account for most of the herds in the temperate regions of Europe, Western Africa and northern Asia. Indicine cattle (*Bos indicus*) are phenotypically identifiable by the presence of a substantial cerciothoracic hump, and are better adapted to arid conditions and dominate across the Indian sub-continent [2].

Today, hybrids of these two types are found throughout sub-Saharan Africa [3], [4]. Regardless of their morphology, however, modern African cattle all possess mitochondrial haplotypes derived from non-humped taurine ancestors [5], [6] although many possess indicine Y-chromosomes [7]. The majority of taurine cattle worldwide are members of mitochondrial macro-haplogroup T, comprising haplogroups T1– T5, although taurine members of haplogroups P, Q and R also exist, albeit at much lower frequencies [8], [9], [10]. It has been shown, however, that while T1, T2 and T4 are monophyletic, T and T3 are not [11]. Nearly all modern African cattle carry haplogroup T1 [6], [8], [12], which is further subdivided into six subhaplogroups T1a – T1f [13]. These subhaplogroups are largely defined by mutations that fall outside the frequently sequenced control region, making the sequencing of complete mitochondrial genomes crucial for advancing our understanding of the phylogeography of T1 cattle in general, and African cattle in particular. Complete mitochondrial genomes of cattle have been published from herds in Egypt and Ethiopia, but nowhere else in Africa [13].

### Movement of Cattle from Northern to Southern Africa

Evidence of domesticated cattle in Africa by about 7,400 years ago includes rock art in what is now the Sahara Desert [14], [15] and archaeological remains attributed to domestic stock based on their bone morphology [3], [16], [17], [18]. Cattle based pastoralism, including dairying [19], was probably practiced throughout northern Africa by about 6,000 years ago and gradually moved south, pushed by desire to escape the increasingly hostile climate of the Sahara [20]. Herders with cattle, sheep and goats spread into eastern Africa (Kenya and Tanzania) shortly before 4,000 years ago and had become widespread across the region by 3,000 years ago [21].

The earliest animal husbandry in southern Africa is associated with transhumant pastoral speakers of Khoisan languages. While recent archaeological research as shown that cattle were present in westernmost South Africa by the early first millennium AD, several hundred years earlier than previously thought [22], whether the spread of domesticates occurred by demic expansion [23], [24] or cultural diffusion [25], [26], [27] remains contentious. Archaeological, genetic and linguistic data do, however, support a model of demic expansion of ethnically and linguistically distinct Bantu-speaking farmers into southern Africa [28]. They carried with them the remainder of the founding population of the region’s cattle, as well as other domestic plants and animals, metallurgy, and ceramics distinct from those of the earlier pastoralists [29], [30], although there is good evidence for interaction with resident foraging groups [31], [32].

The Nguni cattle breed is named for their historical association with speakers of the Nguni language, one of the most southerly members of the Bantu family of languages. We chose to focus on this breed to try to reconstruct the history of cattle expansion because they represent a distinctive, conserved phenotype widely recognized by local farmers, and because there are no modern breeds with direct association with the pastoralists in the region, although there are archaeological specimens of these stock.

## Results and Discussion

We sequenced complete mitochondrial genomes of 35 Nguni cattle on the Roche 454 GS Junior with an average of 37x coverage. We required a minimum of 3 sequences to cover a nucleotide position for calling the consensus and achieved average coverages between 10x and 97x. Sequences have been submitted to GenBank (Accession numbers KF163061-163094). Thirty-two of the samples have the diagnostic transitions diagnostic of haplogroup T1 (16050, 16113 and 16255, cf. the Bovine Reference Sequence (BRS) GenBank Accession number V00654). The remaining three individuals (6, 7, and 8) have 16050 and 16113, but lack 16255. They each, however, carry the transition at 7542 that defines membership in subhaplogroup T1b, so we assume that their lack of 16255 is a consequence of reversion mutations and that they are members of T1.

Thirty-two (91.4%) of the samples are members of subhaplogroup T1b (all except 16, 27 and 40). Four sequences (4, 24, 26 and 32) carry the mutations defining the two subhaplogroups T1b (7542, Ts) and T1c (16122, Ts), however as they all also carry the transversion at 14523 (AT) that defines one of the groups below T1b1 we have assigned them to T1b. All the members of T1b, except one (28) carry the transition at 16022 placing them in T1b1. All the individuals in this study have an insertion, relative to the BRS, at 16201, with three individuals (6, 10, 15) carrying a G and the rest an A. This insertion is not seen in the previously reported complete mitochondrial genomes of African cattle [13]. Of the three individuals not members of T1b, one (40) has the mutations defining T1, but none of the downstream mutations, one (16) is T1c and one (27) is T1d. See Table 1 for the frequencies of the T1 subhaplotypes among our data as well as those previously reported.

**Table 1:** The frequencies of the T1 subhaplogroups found by Bonfiglio et al [13] among Egyptian, Ethiopian and European T1 cattle, tabled with the frequencies among the Southern African Nguni cattle in our dataset.

	Southern African Nguni (n = 35)	Ethiopia (n = 170)	Egypt (n = 26)	Europe (n = 80)
T1	3.0%	0.0%	0.0%	0.0%
T1a	0.0%	25.3%	26.9%	65.0%
T1a	0.0%	25.3%	26.9%	65.0%
T1b	91.4%	69.4%	26.9%	16.3%
T1c	3.0%	0.0%	38.5%	5.0%
T1d	3.0%	5.3%	3.8%	0.0%
T1e	0.0%	0.0%	0.0%	10.0%
T1f	0.0%	0.0%	3.8%	3.7%

**Figure 1:**
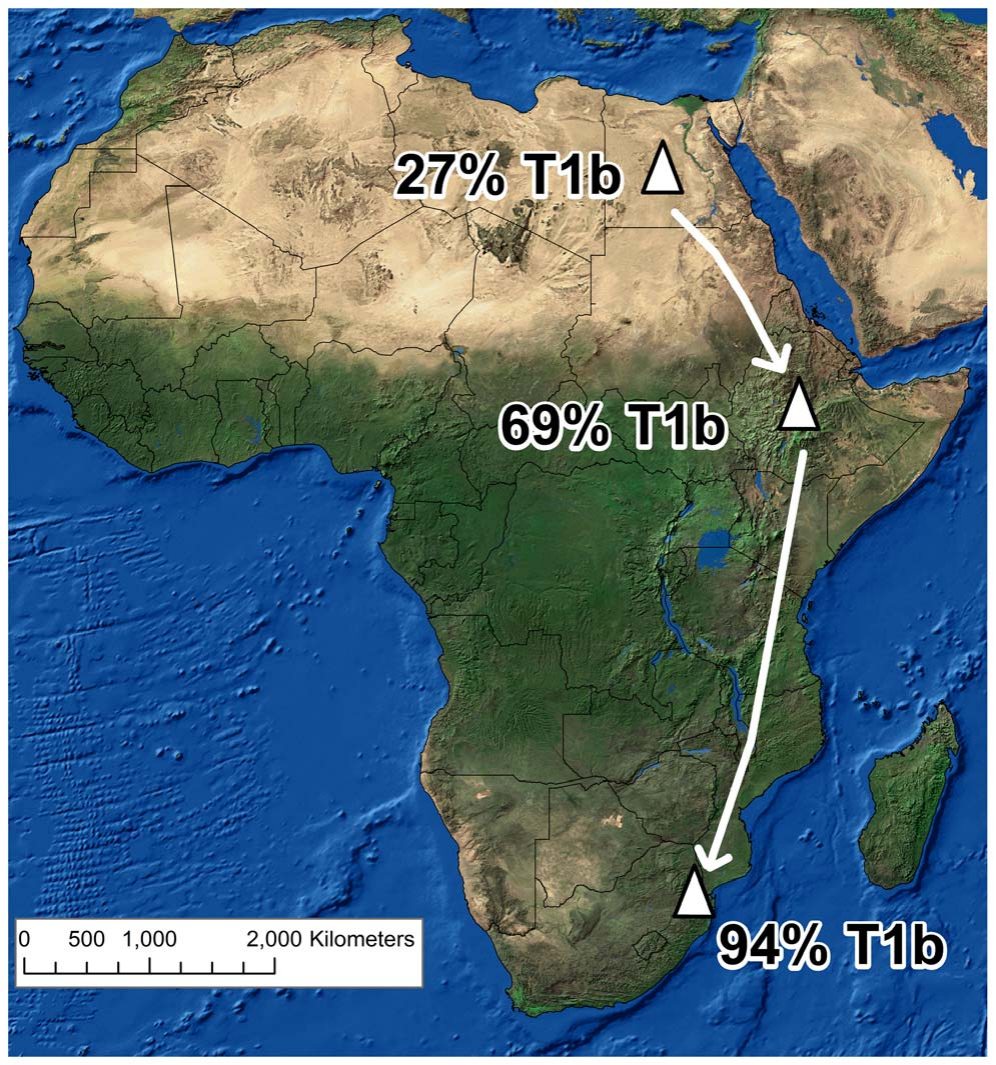
Frequency of subhaplogroup T1b among modern African cattle. Arrows are included only to illustrate the relative order in which populations were established based on radiocarbon dating of archaeological specimens, not as indicators of a specific route of travel [28], [29].

Figure 1 is a median joining network of all available complete mitochondrial genomes of African cattle. It shows that the Nguni sequences are substantially different from those found in Egypt and Ethiopia, and that they display previously uncharacterized variation in T1b1.

We further investigated the demographic signal in the modern mitochondrial genomes of Nguni cattle (Figure S1). In general, our inference supported relaxed clock over a strict clock models with Bayes Factors (BF) of 4.8 to 5. The Bayesian Skyline Plot (BSP) was only marginally supported over the simpler constant population size model (0.08 to 0.19) and thus we were not able to reject the null hypothesis of a constant population size.

**Figure 2:**
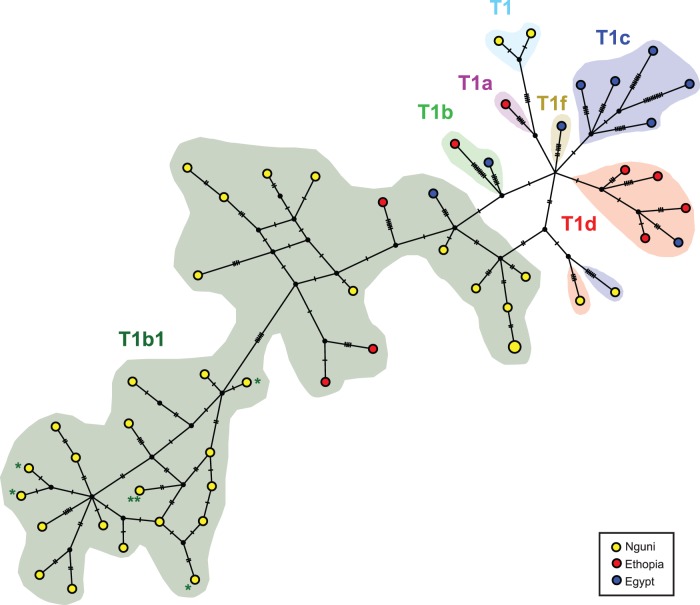
Median-joining haplotype network of modern cattle breeds. Ethiopia (yellow), Egypt (blue) and Nguni (red). Haplotypes are represented by circles. The number of sequences sharing the same haplotype is indicated by the numbers in the ellipses (only numbers bigger than 1 are shown). The different haplogroups were colored coded: T1 (cyan), T1a (purple), T1b (green), T1b1 (dark green), T1c (blue), T1d (red) and T1f (brown). Ethiopian and Egyptian sequences are from Bonfiglio et al. [12]. Nguni sequences are from this study.

The overwhelming majority (91.4%) of the Nguni cattle we studied are members of subhaplogroup T1b. When considered with its modest frequency in Egypt (26.9%) and high frequency in Ethiopia (69.4%), this geographic pattern is consistent with multiple founder effects having substantially shaped the mitochondrial gene pool of southern African cattle (Figure 2). In order to test this hypothesis we first calculated different population genetic statistics. Under serial founder events we expect nucleotide diversity ND) to decrease while pairwise F_ST_ between populations increases with the distance from the source. Our inference shows that nucleotide diversity in the Nguni samples (ND: 0.00075±0.000385) is indeed lower than in Ethiopia (ND: 0.00082±0.000464), which is likewise lower than in Egypt (ND: 0.00094±0.000524). However, the standard errors allow for overlap between the values. Also the pairwise F_ST_ estimation shows a higher differentiation between Nguni and Egypt (F_ST_: 0.463) than Ethiopia (F_ST_:0.437). We found only limited differentiation between Egypt and Ethiopia (F_ST_:0.035). On the contrary to the above discussed pairwise F_ST_ values, which show a p-value below 0.00000, the latter one is non-significant with a value of 0.07207±0.029, probably due to the small sample sizes for Egypt and Ethiopia. It is impossible to say at this stage if this pattern is merely a consequence of isolation by distance, or if other environmental and/or historical factors account for the breeding isolation of founding cattle populations as they spread from north to south. Secondly, we applied model-based Bayesian inference to investigate the split timings between sequences from Nguni, Ethiopia and Egypt. Relaxed molecular clock were supported over a strict molecular clock models with Bayes Factors of about 6 (see Table S1). We did not find stronger support for models with smooth normal prior distributions over ones with fixed mutation rates (see Table S1). Thus, we used the simpler fixed mutation rate model for our inference. Surprisingly, our analysis showed a much younger split (about half the age) between Ethiopia and Egypt (linear model: 8,382(mean) and constant model: 10,446 (mean)) than the split between Nguni and the two other African populations (linear model: 17,086 (mean) and constant model: 19,264 (mean); see Figure S2 and S3). These estimates are older than the suspected timing of cattle domestication and the haplogroup age of T1b [13], but those events are within the 95% highest posterior densities (HPD). In addition, we do have to note that the estimations of split timings, like the BSP expansion timing estimations, are strongly influenced by the mutation rate used and thus have to be interpreted with caution. The inferred younger split between the Egyptian and Ethiopian populations could be explained by population movement among African cattle in recent centuries. The rinderpest panzootic in the 1890s killed the majority of the cattle in sub-Saharan Africa [33], and we could reasonably expect a repopulation of the eastern Africa from north Africa as well as substantial population movement, which could account for the patterns seen. The inferred tree topology does not exclude serial founder events, as discussed above, but does indicate a much younger cattle movement into eastern Africa.

In taking on this study we realized there was a chance that local hybridization might have obliterated any chance for reconstructing the origins of the breed. Archaeological evidence [29], [34], [35], as well as data from the autosomes and sex chromosomes of African cattle [36], [37], [38], [39] indicate significant trade, including cattle, coming into southern Africa from the Indian subcontinent. Our data, however, are consistent with previous studies [5], [6], [13] showing that the Indian Ocean trade had no impact on the maternal inheritance of African cattle.

## Methods and Materials

### Ethics Statement

This study was reviewed and approved by the Otago University Animal Ethics Committee.

### Samples

The 35 Nguni cattle sampled in this study were members of a herd farmed by Cedric Stoch at Droërug Farm, Malmesbury, South Africa, and they were sampled with his permission. Mr. Stoch is an active member in good standing of the Nguni Cattle Breeders Society of South Africa. Stoch’s herd was originally sourced from five herds across Swaziland. In July 2012, he pulled hair from the tails of his cattle and stored them in ethanol. DNA was extracted with the Qiagen DNeasy animal extraction kit following the manufacturer’s protocol.

### Amplification

When the quality of the extracted DNA allowed, the complete mitochondrial genome was PCR amplified in two overlapping fragments, using primer pairs Bos510 and Bos 535, and Bos 534 and Bos511 (see Table S2 for primer sequences and Table S3 for fragment lengths). Long range PCR was undertaken using Roche’s Expand Long Range dNTPack and the following reagent concentrations: 1x Long Range Buffer, 0.4 µM of each primer, 0.4 mM of each dNTP, 3% (v/v) DMSO (dimethyl sulfoxide) and 2 units of enzyme. 3 µL of DNA extract was added for a total reaction volume of 30 µL. PCR conditions consisted of an initial denaturation period of 92°C for 2 minutes, followed by 10 cycles of 92°C for 10 seconds, 55°C for 10 seconds and 68°C for 8 minutes and 30 seconds. This was followed by 20 cycles of 90°C for 10 seconds, 55°C for 15 seconds and 68°C for 8 minutes and 30 seconds, with an increase 20 seconds at 68°C each cycle. The cycling ended with 68°C for 7 minutes. PCR products were visualized under UV on SYBR Safe (Invitrogen) stained 2% agarose gels. The samples that could not be successfully amplified in two fragments, were amplified in three or four with primers Bos 518, Bos 519, Bos 548, Bos 549 (see Table S2 for primer sequences and Table S3 for fragment lengths).

PCR products were purified using Qiagen’s QIAquick PCR purification kit according to the manufacturer’s instructions, and quantified using a Nanodrop spectrophotometer.

### Fragmentation and Barcoding

PCR products from each individual were pooled in equimolar ratios, and 1 µg of DNA was fragmented with the NEGNext dsDNA Fragmentase according to the manufacturer’s protocols and an incubation period of 18 minutes. DNA barcoding was undertaken using the protocol described in Meyer et al [40].

### 454 DNA Sequencing

Equimolar amounts of all 35 barcoded mitochondrial genomes were mixed together to form a single pooled sample and used to construct a Rapid DNA Sequencing Library (454 Life Science/Roche) as per manufacturer’s instructions except that the sample was not nebulized prior to fragment end repair. Library quality was confirmed from a Bioanalyzer trace (Agilent Technologies) and the concentration in molecules per microliter determined by comparing a fluorescent label incorporated into one of the library adaptors to a standard curve using a Victor X fluorescence plate reader (PerkinElmer). This library was used in an emulsion PCR at a ratio of 3cpb using the GS Junior Titanium Series Chemistry (454 Life Sciences/Roche). All of the enriched beads were used for sequencing on the Roche/454 Life Sciences GS Junior instrument according to the manufacturer’s instructions.

### Raw Sequence Data Processing

The reads were first sorted according to their barcode using the freely available software tool “untag” (https://bioinf.eva.mpg.de/pts/). In the next step the tagcleaner software (Schmieder et al. 2010; http://tagcleaner.sourceforge.net) was used to remove long-range priming sites. Then the reads were mapped against a reference sequence (complete mitochondrial genome of *Bos taurus* GenBank number V00654) using the Burrows–Wheeler Alignment Tool (BWA; [41]). The created sam files were then processed with the software tool samtools [42] to call consensus sequences and variants such as single nucleotide polymorphisms (SNPs; using bcftools, which is part of the samtools package). The sequence data quality was checked using FastQ (http://www.bioinformatics.babraham.ac.uk/projects/fastqc/. Accessed 2013 Feb 7).

#### Demographic inference

In order to reconstruct the demographic history of Nguni cattles and relationships between African cattle populations we used model-based Bayesian inference as implemented in BEAST2.0.2 [43]. We performed Bayesian Skyline plot (BSP) analyses of the 35 complete mitochondrial genomes from the Nguni cattle using BEAST2.0.2 [43], [44].The Markov chain Monte Carlo (MCMC) chains were run for 100,000,000 iterations each, sampling every 10,000th step and a burn-in of 10,000,000 steps. In order to test the support of relaxed versus strict molecular clock models and the BSP versus a simpler constant population size prior (10000–5,000,000; same MCMC run properties as the BSP analysis), we estimated the log_10_ Bayes factor using TRACER 1.5 [45]. TRACER 1.5 was also used to verify effective sampling size (ESS) scores and the trace of the MCMC runs as well as to visualize the inferred demographic changes. To investigate relationships between African cattle populations in a model-based framework we used *BEAST [46] to reconstruct the phylogenies under a linear with constant root demographic model and a constant size model. We ran *BEAST using a relaxed and a strict molecular clock as well as models with fixed and smooth normal priors to estimate mutation rates. We applied a reversible jump based substitution model selection using the BEAST2.0.2 add-on RBS (http://beast2.cs.auckland.ac.nz/index.php/Add-ons) in the *BEAST as well as in the Nguni demography analyses. The trees were summarized and displayed using TreeAnnotator (part of the BEAST2 package ), Figtree (http://tree.bio.ed.ac.uk/software/figtree/) and DensiTree (part of the BEAST2 package ). The mean estimates of the female effective population sizes along the tree were extracted from the *BEAST log file using the Python script *starbeast_demog_log.py* from the biopy package (http://code.google.com/p/biopy/).

### Biogeographic Reconstructions

We reconstructed a median-joining [47] haplotype network of the Nguni sample and the samples from Egypt and Ethiopia [13]using the software PopART (http://leigh.geek.nz/software.shtml).

## Supporting Information

Figure S1 **Demographic history of Nguni cattle.** (A) Reconstructions using a relaxed molecular clock. Left: Constant size prior and Right: Bayesian Skyline Plot. (B) Reconstructions using a strict molecular clock. Left: Constant size prior and Right: Bayesian Skyline Plot.
(EPS)

Figure S2 **Model-based inference of population relationships in African cattle using a linear with constant root population size model.** (A) Posterior consensus tree. Blue beams indicate 95% HPD intervals of the respective split timing. Mean estimates are provided at the respective nodes. (B) Summary of posterior trees (blue) generated using DensiTree (part of the BEAST2 package). Green lines indicate female effective population size changes over time projected onto the tree. Mean estimates are provided. Italic numbers indicate Bayesian branch support.
(EPS)

Figure S3 **Model-based inference of population relationships in African cattle using a constant population size model.** (A) Posterior consensus tree. Blue beams indicate 95% HPD intervals of the respective split timing. Mean estimates are provided at the respective nodes. (B) Summary of posterior trees (blue) generated using DensiTree (part of the BEAST2 package). Green lines indicate female effective population size changes over time projected onto the tree. Mean estimates are provided. Italic numbers indicate Bayesian branch support.
(EPS)

Table S1 Bayes Factors (BF) and marginal likelihoods for the *BEAST analyses using different priors. Relaxed molecular clock: [1] fixed mutation rate and [2] prior using a normal distribution. Strict molecular clock: [3] fixed mutation rate and [4] prior using a normal distribution. Relaxed molecular clock: Constant size prior ([A] fixed mutation rate and [B] prior using a normal distribution) and linear with constant root prior ([C] fixed mutation rate and [D] prior using a normal distribution).
(DOCX)

Table S2 **PCR primer sequences.**
(DOCX)

Table S3 **PCR fragment lengths.**
(DOCX)
