# Supplementary material for: The Genetic Diversity of the Nguni Breed of African Cattle (Bos spp.): Complete Mitochondrial Genomes of Haplogroup T1
Source: PLoS One. 2013 Aug 19;8(8):e71956. doi: 10.1371/journal.pone.0071956 (PMC3747060; doi:10.1371/journal.pone.0071956)
Supplement: Table S1 — Bayes Factors (BF) and marginal likelihoods for the *BEAST analyses using different priors. Relaxed molecular clock: [1] fixed mutation rate and [2] prior using a normal distribution. Strict molecular clock: [3] fixed mutation rate and [4] prior using a normal distribution. Relaxed molecular clock: Constant size prior ([A] fixed mutation rate and [B] prior using a normal distribution) and linear with constant root prior ([C] fixed mutation rate and [D] prior using a normal distribution). (DOCX) [file pone.0071956.s004.docx]

**Table S1 - Bayes Factors (BF) and marginal likelihoods for the *BEAST analyses using different priors.** Relaxed molecular clock: [1] fixed mutation rate and [2] prior using a normal distribution. Strict molecular clock: [3] fixed mutation rate and [4] prior using a normal distribution. Relaxed molecular clock: Constant size prior ([A] fixed mutation rate and [B] prior using a normal distribution) and linear with constant root prior ([C] fixed mutation rate and [D] prior using a normal distribution).

**Linear with Constant Root Prior**

|  | **mutation rate** | **marginal likelihoods** | **BFs**  **[1]** | **[2]** | **[3]** | **[4]** |
| --- | --- | --- | --- | --- | --- | --- |
| **Relaxed Clock [1]** | fixed | -23951.93 | - | 0.96 | 6.11 | 5.87 |
| **[2]** | normal distribution | -23954.14 | -0.96 | - | 5.15 | 4.91 |
| **Strict Clock [3]** | fixed | -23966 | -6.11 | -5.15 | - | -0.23 |
| **[4]** | normal distribution | -23965.46 | -5.87 | -4.91 | 0.23 | - |

**Constant Size Prior**

|  |  | **mutation rate** | **marginal likelihoods** | **BFs**  **[1]** | **[2]** | **[3]** | **[4]** |
| --- | --- | --- | --- | --- | --- | --- | --- |
| **Relaxed Clock [1]** | **Relaxed Clock** | fixed | -23956.34 | - | -1.93 | 3.79 | 4.85 |
| **[2]** |  | normal distribution | -23951.90 | 1.93 | - | 5.73 | 6.78 |
| **Strict Clock [3]** | **Strict Clock** | fixed | -23965.08 | -3.79 | -5.73 | - | 1.06 |
| **[4]** |  | normal distribution | -23967.51 | -4.85 | -6.78 | -1.06 | - |

**Constant Size versus** **Linear with Constant Root Prior (Relaxed Clock)**

|  | **mutation rate** | **marginal likelihoods** | **BFs**  **[A]** | **[B]** | **[C]** | **[D]** |
| --- | --- | --- | --- | --- | --- | --- |
| **Constant**  **[A]** | fixed | -23956.34 | - | -1.93 | -1.92 | -0.96 |
| **[B]** | normal distribution | -23951.90 | 1.93 | - | 0.01 | 0.973 |
| **Linear**  **[C]** | fixed | -23951.93 | 1.92 | -0.01 | - | 0.96 |
| **[D]** | normal distribution | -23954.14 | 0.96 | -0.97 | -0.96 | - |
