# Supplementary material for: The Genetic Diversity of the Nguni Breed of African Cattle (Bos spp.): Complete Mitochondrial Genomes of Haplogroup T1
Source: PLoS One. 2013 Aug 19;8(8):e71956. doi: 10.1371/journal.pone.0071956 (PMC3747060; doi:10.1371/journal.pone.0071956)
Supplement: Table S2 — PCR primer sequences. (DOCX) [file pone.0071956.s005.docx]

**Table S2**. **PCR primer sequences.**

| **Primer Name** | **Primer Sequence** |
| --- | --- |
| Bos 510 | TCGTAGGGCTCCGATTAGTG |
| Bos 511 | GATCAGGCTGAGCTTCCAAC |
| Bos 518 | GGGGTGTCCAAAGAATCAGA |
| Bos 519 | AACAGGCTGAACCGTGTACC |
| Bos 534 | GGGGCTTCTACGTGAGCTTT |
| Bos 535 | AACGCCGGACTCTATTTCCT |
| Bos 548 | TGCTAGTTGTCCGATGGTGA |
| Bos 549 | AATTGGAGGACAACCAGTCG |
