# Supplementary material for: The Genetic Diversity of the Nguni Breed of African Cattle (Bos spp.): Complete Mitochondrial Genomes of Haplogroup T1
Source: PLoS One. 2013 Aug 19;8(8):e71956. doi: 10.1371/journal.pone.0071956 (PMC3747060; doi:10.1371/journal.pone.0071956)
Supplement: Table S3 — PCR fragment lengths. (DOCX) [file pone.0071956.s006.docx]

**Table S3. PCR fragment lengths.**

| **Primer Pair** | **Fragment Length (bp)** |
| --- | --- |
| Bos 510 – Bos 535 | 8873 |
| Bos 534 – Bos 511 | 7747 |
| Bos 510 – Bos 549 | 4315 |
| Bos 548 – Bos 535 | 4610 |
| Bos 511 – Bos 518 | 2957 |
| Bos 519 – Bos 534 | 5145 |
